# Concordance of three point of care testing devices with clinical chemistry laboratory standard assays and patient-reported outcomes of blood sampling methods

**DOI:** 10.1186/s12911-022-01999-z

**Published:** 2022-09-22

**Authors:** Z. Yonel, K. Kuningas, P. Sharma, M. Dutton, Z. Jalal, P. Cockwell, J. Webber, P. Narendran, T. Dietrich, I. L. C. Chapple

**Affiliations:** 1 The Periodontal Research Group, School of Dentistry, University of Birmingham, 5 Mill Pool Way, Birmingham, B5 7EG UK; 2 Department of Renal Medicine, University Hospitals Birmingham NHS Foundation Trust, Queen Elizabeth Hospital Birmingham, Mindelsohn Way, Edgbaston, Birmingham, B15 2GW UK; 3 School of Pharmacy, University of Birmingham, Birmingham, UK; 4 Diabetes Unit, University Hospitals Birmingham NHS Foundation Trust, Queen Elizabeth Hospital Birmingham, Mindelsohn Way, Edgbaston, Birmingham, B15 2GW UK

**Keywords:** Point of care testing, Screening, Prevention

## Abstract

**Background:**

Point of care testing (POCT) devices have been developed to facilitate immediate results with the potential to aid screening for new disease and enable patients to self-monitor their disease. Non-communicable diseases (NCDs) are the major cause of mortality globally and are increasing in prevalence as the population ages. Allied health care professionals (AHPs) are skilled in undertaking risk assessment and delivering preventative advice, providing opportunities to access large proportions of the population who may not visit their doctor, within non-traditional community settings. There is evidence of high levels of support from public, patients and health professionals for engaging AHPs in risk-targeted early case detection of certain NCDs. Thus, POCT devices offer a potential alternative to traditional venous blood collection, as novel care pathways for increasing early case detection and access to preventative care. The objectives of this study were to: (i) determine the concordance of the specific POCT devices with laboratory-based standard assays employed within clinical biochemistry laboratories. (ii) compare the sampling experience of both methods via patient-reported experiences.

**Methods:**

A prospective, two-centre study was undertaken involving 158 participants who provided informed consent. Venous blood was collected for traditional assays of HbA1c, creatinine/ estimated Glomerular-Filtration-Rate (eGFR) and vitamin-D. Capillary blood was collected by finger prick test and also assayed for the same biochemical indices (Nova StatSensor (creatinine/eGFR); Siemens DCA-Vantage (HbA1C); CityAssays (vitamin-D)). All users were provided with device training. Participants reported any discomfort experienced by each simultaneously applied method (randomised in order) via a 100 mm Visual-Analogue-Scale.

**Results:**

Results for each POCT device and the laboratory standard were analysed by Bland-Altman plots to determine assay concordance. POCT devices demonstrated good concordance with laboratory testing, with at least 95% of all samples being within two standard deviations, for each of the devices tested. The majority of participants reported less discomfort with POCT than venepuncture, with the average reported discomfort being 17/100 mm less for POCT compared to venous blood sample collection on the visual analogue scale.

**Conclusions:**

The POCT devices demonstrated acceptable concordance with laboratory-based assays, and patients reported lower levels of discomfort compared to traditional means of blood collection. This study demonstrates the potential of using these devices as acceptable methods for opportunistic testing of “at-risk” individuals within non-traditional community care settings.

## Background

The prevalence of non-communicable diseases (NCDs) is increasing worldwide. This has a significant impact on the global disease burden and healthcare economy. The impact of the major NCDs (diabetes, cardiovascular diseases, cancer, chronic respiratory diseases and mental disorders) account for an estimated 86% of the deaths and 77% of the disease burden in Europe [1]. The increasing prevalence of NCDs is, in part, attributed to an increasingly ageing population, but also due to an increase in the prevalence of risk-factors common amongst most NCDs such as physical inactivity, refined diets and overweight/obesity. In 2011 the United Nations General Assembly received the commitment from world leaders to take measures to tackle NCDs. Subsequently there have been several policy interventions to support this agenda. Notably the inclusion of NCDs, with measurable targets and indicators, under the third of the “Sustainable Development Goals” [2].

The incidence of NCDs is a key example of the health inequalities that pervade modern society, as lower socio-economic groups struggle to access preventative services due to cost and geographic location. In addition to the substantial health burden, NCDs also contribute a significant economic burden. A report published by the World Economic Forum and the Harvard School of Public Health in 2011, predicted that over the next 20 years, NCDs will cost more than 30 trillion US$, which is the equivalent of 48% of global GDP in 2010. The report goes on to state that lost output from the five most prevalent NCDs over the period 2011–2030 is estimated at nearly 47 trillion US$ [3]. Furthermore, in Europe in 2015, public expenditure on health was 7.8% of GDP in the EU as a whole and in 2013, premature deaths due to major NCDs cost EU economies around 0.8% of GDP. Moreover, non-health costs of NCD in the EU such as productivity losses due to mortality and morbidity associated with CVD cost €54 billion in 2015 alone [4].

Given the growing NCD burden and the fact that allied health professionals (AHPs) have access to large proportions of the population who may not engage with other healthcare services [5], AHPs are ideally placed to assist with the early identification of NCDs in non-traditional community settings. Dental care professionals (DCPs) and pharmacists are trained and skilled in risk-assessment and routinely deliver preventative advice, such as smoking cessation, exercise and advice on healthy nutrition. Risk assessment for specific NCDs, followed by early case detection is a natural extension of their current roles. Importantly, stakeholder opinion for AHPs undertaking risk assessments for certain NCDs is extremely positive [5, 6]. Public support for screening for medical conditions in both dental and pharmacy settings is strong, with particular support for risk-targeted early case detection in type 2 diabetes (T2DM) and hypertension [5]. Patients as well as pharmacists, physicians and dentists also support public opinion for such novel care pathways [7].

Point of care testing (POCT), is a testing method that does not require samples to be sent to an accredited laboratory, and instead is undertaken near the patient, often chairside or bedside and provides results in a short timeframe [8]. POCT can be of benefit when an immediate result is required or when access to a laboratory is not feasible, practical or readily available. This may be the case in community-based healthcare settings such as primary care dental practices and community pharmacy settings.

The National Institute for Health and Care Excellence (NICE) in England currently recommends that AHPs risk-assess for T2DM [9]. Data from the US and Europe suggest that screening for T2DM in a dental setting is effective for identifying those at high risk and those who already unknowingly have the condition [6, 10–15]. Whilst NICE guidance currently suggests using a validated risk assessment questionnaire, the literature suggests POCT devices are often used in conjunction or instead of these validated questionnaires [6, 10, 14–17].

UK government policies actively encourage dental care professionals (DCPs) to deliver general health promotion [18, 19]. It has been suggested that highly skilled primary healthcare professionals, such as DCPs, may develop new roles and integrate care provision more seamlessly with GPs to create effective multi-disciplinary teams and care-pathways to benefit patients. The provision of a wider range of services by AHPs in collaboration with GPs, such as early detection of systemic NCDs, provides greater access to care for vulnerable groups and helps to address the highly prevalent healthcare inequalities that have been highlighted by the SARS-COV-2 pandemic [20, 21]. This aligns closely with the UK “Making Every Contact Count” agenda to improve general health and wellbeing [22]. Similarly, UK policy and pharmacists' professional organisations have stressed the potential of community pharmacists to extend their roles in patient care services to include screening for NCDs. This has been emphasised in policy papers calling for a wider use of community pharmacists in primary patient care [20, 23, 24]. In the UK, POCT is considered an important development area for the future of pharmacy, it is supported by the Royal Pharmaceutical Society and the National Pharmaceutical Association. Several pilot initiatives in pharmacies across the UK have taken place, including testing for T2DM, coronary heart disease and cholesterol [25].

This study forms part of a broader body of work to determine: the acceptability to stakeholders (patients, the public, and healthcare professionals) of utilising AHPs to undertake risk-targeted early case detection of potentially high-risk individuals for specific non-communicable diseases (NCDs) [5], patient acceptability of undertaking risk-targeted early case detection for NCDs within a general dental practice and community pharmacy setting [6, 26], and the concordance of point of care testing (POCT) devices against laboratory methods.

Controversies surrounding the reliability and accuracy of POCT devices has traditionally provided a barrier to their uptake [27, 28], as has the variability in precision of the large number of available devices [29]. Venous blood analysis using laboratory-based methods remains the reference standard. However, the improved quality and precision of POCT devices for capillary blood sampling has led to NICE and other national bodies recommending their use for diagnosis in some cases [27, 30–33]. Despite this recommendation, the preference for conventional diagnostic methods by a physician for formal diagnosis and appropriate provision of treatment plans remain. Given that venous sample collection in many community settings is challenging it is important that POCT devices if utilised demonstrate high concordance with current standard reference-assays.

Here we report a two-staged exploratory study. Stage one aimed to measure the agreement between POCT devices with a central laboratory method for: HbA1C (diabetes), creatinine/e-GFR (chronic kidney disease) and total vitamin D. The devices calibrated were the Nova StatSensor (creatinine/estimated Glomerular-Filtration-Rate [eGFR]), Siemens DCA Vantage (HbA1c), and CityAssays (vitamin D capillary blood-spot tests). This stage also aimed to gauge the opinions of the participants regarding acceptability of the method of blood collection. Stage two comprised a study within one dental and one community pharmacy in the West-Midlands, UK, to determine patient acceptability of risk-assessment for NCDs in these settings, utilising validated risk-questionnaires followed by the POCT devices [26].

## Methods

This was a prospective study of 158 volunteers, recruited from the Queen Elizabeth Hospital (QEH) and Birmingham Dental Hospital (BDH). Ethical approval was obtained from South East Scotland Research Ethics Committee (REC reference: 16/SS/0197) and informed written consent obtained from each participant. All methods were carried out in accordance with relevant guidelines and regulations.

### Inclusion & exclusion criteria

*Inclusion criteria*

a.Aged > 18 years.

b.Willing and able to provide valid informed consent.

c.Attend outpatients’ departments at Queen Elizabeth Hospital, Birmingham (QEH) or Birmingham Dental Hospital (BDH).

*Exclusion criteria*

a.Aged < 18 years.

b.Unable or unwilling to provide valid informed consent.

### Recruitment

Consecutive patients meeting the study eligibility criteria and attending outpatient appointments at the QEH and BDH were approached by a member of study team and offered the opportunity to participate. If the patient was interested, further information relating to the study including the patient information leaflet was provided by a study team member trained in consent.

### Blood collection

Venous blood samples were collected alongside the patient’s routine care requirements and sent to the Clinical Chemistry laboratory at University Hospital Birmingham Foundation Trust’s QEH for assay. The time of blood collection and testing by both capillary and venepuncture methods was recorded to ensure they were within 15 min of each other. In this study all patients received their venous blood sample collection first, followed by POCT.

The laboratory methods employed at the QEH Clinical Chemistry Laboratory were: the TOSOH G8 High Performance Liquid Chromatography (HPLC) for HbA1c measurement. Serum creatinine was measured by Alinity c enzymatic method. All blood collection methods being subject to external accreditation by UKAS against ISO15189 for quality assurance.

Finger-prick (capillary) testing was performed according to standardised operating procedures (SOP), in accordance with manufacturer’s guidelines and study protocols. The same brand and gauge lancet was used for each participant in order to draw blood.

### Nova StatSensor

An analytical method correlation was performed using discarded whole blood lithium heparin samples. Quality control (QC) tests were performed daily for each POCT device as per device protocols. Fifty-three patients attending for outpatient appointments at the QEH with different stages of chronic kidney disease (CKD) were asked to contribute a StatSensor finger-prick sample for serum creatinine at a routine visit at the renal clinic, where formal kidney function testing was also undertaken. Twelve patients with eGFR ≤ 20 were recruited, 13 patients with eGFR 20–29, 15 patients with eGFR 30–44 and 13 patients with eGFR 45–59. Each sample was processed in accordance with manufacturers guidelines.

### DCA vantage and city assays

No prior calibration of equipment was required for either the CityAssays or DCA Vantage POCT. Fifty participants were recruited at Birmingham Dental Hospital and consented for a finger-prick blood spot CityAssay vitamin D test and a venous (control) blood sample. The capillary vitamin D test required the capillary test strip to be mailed to the laboratory for assay with results returned to both the patient volunteer (via an online reporting platform) and study team directly and within 3 working days. One participant’s sample was deemed insufficient to provide a result; the remaining forty-nine results were analysed.

Fifty-six T2DM patients with different levels of glycaemic control were asked to contribute a finger-prick sample at a routine outpatient visit at the QEH, where routine HbA1c testing on a venous blood sample was also undertaken. Systemically healthy controls (n = 10) were also recruited at BDH for the lower end of the calibration line. Each sample was processed in accordance with manufacturers’ guidelines.

### Visual analogue score (VAS)

Each participant who consented to undergo finger-prick testing was also asked to complete a Visual Analogue Scale to assess the perceived discomfort related to that experience [34]. Discomfort was recorded at the time as well as the residual level of discomfort they felt “some time” later (5 min -15 min post-sample collection).

### Primary outcome

The primary outcome of interest was the concordance of results from the capillary POCT with the laboratory tested venous sample for identifying creatinine, HbA1C and vitamin D levels.

### Data analysis

The percentage bias of each POCT result compared to the laboratory reference result was calculated and analysed using a Bland Altman plot in order to assess accuracy [35]. Descriptive statistics were also used to analyse data and for the VAS. Wilcoxon Signed-rank Test was used to compare VAS results for finger-prick and venous blood sample. A subgroup analysis was undertaken, as some of the cohorts were familiar with either venous blood samples, due to regular visits to outpatient services, or finger-prick testing amongst the T2DM cohort due to regular home testing. The sensitivities and specificities for each POCT device were also calculated comparing the finger-prick sample to the venous reference standard.

## Results

Overall, the data demonstrated that the POCT devices used for HbA1c, Creatinine and Vitamin D testing were comparable to the current reference-standard venous blood sample assays, with strong levels of concordance. Patients reported that POCT was an acceptable method of blood collection, generally being less uncomfortable than traditional venous blood tests at the time of sample of collection.

### Nova StatSensor [POCT creatinine]

**Fig. 1:**
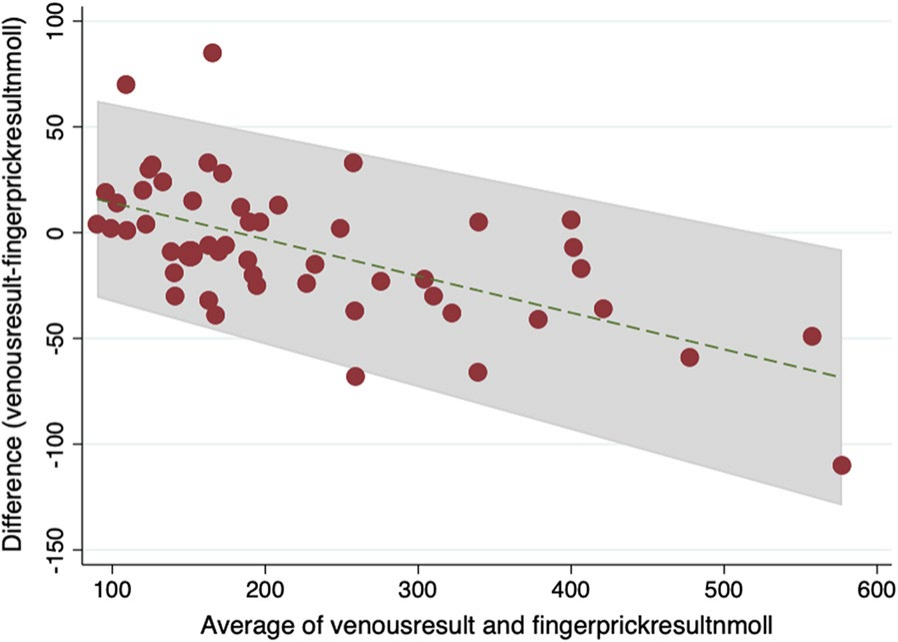
BA plot comparing venous sample with POCT creatinine results in nmol/L

A Bland–Altman (BA) plot was used to compare the creatinine concentration measured within the standard venous sample and that obtained with the POCT device [Fig. 1]. The BA plot demonstrates that 50/52 results were within two standard deviations of the mean difference between assays, indicating that the two methods could be used interchangeably [35, 36]. Despite the results showing good concordance, the BA plot for creatinine does indicate a possible proportional bias, whereby for those patients who have a low creatinine, the POCT finger prick sample gives a lower result than the venous sample (reference standard) and for higher creatinine, the POCT finger prick samples are higher than the venous sample. POCT had 98.8% sensitivity (95% CI 95.6: 99.9) and 100% specificity (95% CI 29.2: 100) for a reference standard test outside the reference range.

### Siemens/Bayer DCA vantage

**Fig. 2:**
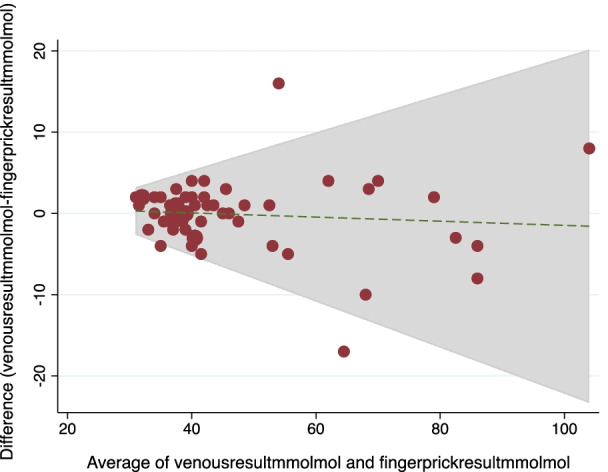
BA plot comparing venous sample with POCT HbA1C results in mmol/mol

The BA plot demonstrates that 53/56 results are within two standard deviations of the mean difference between the methods, indicating acceptable levels of comparability [Fig. 2]. The POCT device showed a sensitivity of 87.5% (95% CI 67.6: 97.3) and specificity of 84.4% (95% CI 67.2: 94.7).

### CityAssays

**Fig. 3:**
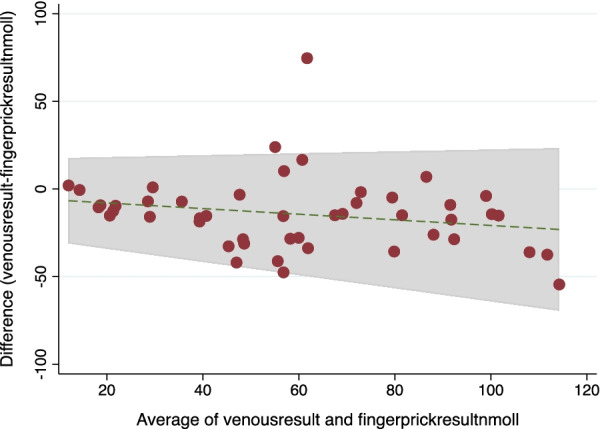
BA plot comparing venous sample with POCT Vitamin D results in nmol/L

The BA plot shows that 48/49 results were within two standard deviations of the mean difference between the methods, suggestive that the two tests are comparable [Fig. 3]. POCT device showed a sensitivity of 91.3% (95% CI 72: 98.9) and specificity of 61.5% (95% CI 40.6: 79.8).

### Visual analogue scores (VAS)

Discomfort as a result of the procedure was recorded at two timepoints; at the time of procedure and residual discomfort after the procedure (5–15 min) for both the POCT and venous blood samples. Wilcoxon Signed-rank Test comparing of VAS results for finger-prick and venous blood sample revealed the two testing methods to be comparable in relation to patient comfort.

**Fig. 4:**
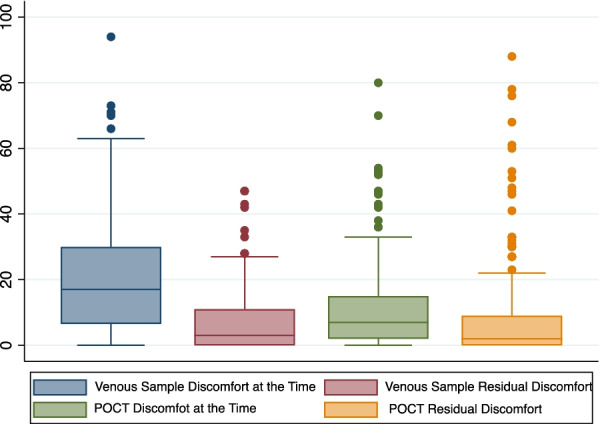
Box and Whisker plot demonstrating discomfort at time and after sampling method

Overall, the median pain scores with venous blood sampling 17/100 were significantly higher than the median pain scores with a finger-prick test 7/100. When asked at time of testing, people experienced more discomfort with the venous blood test compared with a finger-prick test, with the venous blood test scoring 9/100 higher than finger prick testing. Whereas on average people found the venous blood sample and finger-prick testing to be comparable, for residual pain after the sampling procedure, with an average difference in score of 0 points out of a hundred [Fig. 4].

### Sub-groups

*Patients accustomed to venous blood tests*, such as patients with CKD (n = 52), on average experienced more discomfort at the time of testing with the venous blood sample scoring on average 11/100 more than finger-prick testing. Whereas they found venous blood testing comparable (0/100 difference) to finger-prick testing in terms of residual pain post-procedure.

*Patients accustomed to finger-prick testing*, such as patients with diabetes (n = 56), on average also experienced more discomfort at time of testing with the venous blood sample scoring on average 10/100 more than finger-prick testing. This patient group also found venous blood testing to be broadly comparable to finger-prick testing in terms of residual pain after the procedure, with venous blood sample being on average only 1/100 greater than finger prick testing.

*Patients accustomed to neither finger-prick nor venous sampling (n* = *49)*, such as patients likely to access dental and pharmacy settings, on average experienced more discomfort with the venous blood score at the time of procedure of 4/100 more than with a finger-prick test at the time of the testing. This group also found venous blood testing comparable to finger prick testing in terms of residual pain after the procedure.

## Discussion

This study has demonstrated that the POCT devices evaluated for HbA1C, creatinine and vitamin D testing were comparable to current laboratory-based assays used in day-to-day hospital practice. Level of discomfort reported by patients was comparable overall for both methods of blood sample collection, finger-prick and venous. Finger-prick testing was identified as an acceptable method of testing for the majority of participants and deemed less uncomfortable than venous sampling by the majority of participants at time of sample collection [Table 1].

**Table 1:** Table showing descriptive statistics relating to reported discomfort according to a 100 mm visual analogue scale

	Number of observations	Mean	Median	Standard deviation	Minimum value	Maximum value
Conventional venous sample at the time of procedure	164	30.0	17	18.8	0	94
Conventional venous sample—residual symptoms	164	7.0	7	9.7	0	47
POCT Finger-prick at time	164	12.0	7	15.0	0	80
POCT Finger-Prick—residual symptoms	164	9.2	2	16.7	0	88
Difference between venous sample at the time of procedure & POCT Finger-prick at time	164	9.0	6	18.2	-53	83
Difference between conventional residual symptoms & POCT Finger-Prick Residual symptoms	164	2.2	0	17.1	83	39

Historically, controversies surrounding the reliability and accuracy of POCT devices have been a barrier to their use in detection and diagnosis of NCDs [27]. Likewise, the variability in precision of the large number of devices available has also impacted on the uptake of POCT [28, 29]. However, this study has demonstrated that despite the controversies surrounding POCT, in particularly in relation to a perceived lack of accuracy, reliability and concerns relating to interpretation of results, the devices used in this study demonstrated high levels of concordance with conventional laboratory-based assays of venous blood. All three devices showed good concordance with results being within two standard deviations of the mean difference between the methods, indicating acceptable levels of comparability. Furthermore, all three tests showed specificities > 80%, thus were reasonable at identifying those who do not have the target condition. The sensitivity for both creatinine and HbA1c were also greater than 80%. As AHPs should not use POCTs for diagnosis but more risk-targeted early case detection, this level of accuracy would be sufficient to identify those who may benefit from follow up with their healthcare provider. Furthermore, the POCT would supersede the accuracy of conventional risk-prediction models available for these target conditions, thus potentially streamlining the onward management process and limiting unnecessary referrals to primary care colleagues.

The results for the DCA vantage POCT for testing HbA1c levels showed both a sensitivity and specificity > 80% and concordance with the reference standard. A recent study comparing 7 POCT devices for HbA1c found only 4 instruments met the generally accepted performance criteria for HbA1c, of which the DCA vantage was one [37]. However, a systematic review and meta-analysis released recently has urged caution in use of POCT devices when used for diagnosis [38]. Nine of the devices considered, including the DCA Vantage showed potential for a negative bias which may lead to under diagnosis. However, a meta-regression was used to explore temporal effects and demonstrated the precision of the DCA vantage improved over time. In the meta-regression studies were dichotomised into those prior to 2006 and those from 2006 to 2016. The results suggested a significant reduction in bias within those studies undertaken post 2006 compared to those studies prior to 2006 [38]. The DCA vantage was one of two devices to show no difference in bias between clinical or laboratory operators, thus suggesting reduced technique sensitivity and ease of use in the clinical setting [38]. This is an important consideration if the device is to be considered for used by AHPs in community settings.

A recent study comparing two POCT devices for assessment of renal function, one of which was the Nova StatSensor, reported that the POCT devices were only moderately accurate at detecting renal impairment in patients undergoing radiological investigations, but seemed to be a good screening tool. The study recommended, any low eGFR (≤ 30) values should be further examined due to the under-reporting of eGFR values in some cases, although the POCT devices did not actually miss any high-risk patients [39].

In our study we found the Nova StatSensor to have the highest sensitivity and specificity of the three POCT devices assessed, and it showed good concordance with the reference standard as demonstrated via the Bland–Altman plot. A further study evaluating the Nova StatSensor reported that it showed results that were “acceptable-to-good” in terms of repeatability, inter-device reproducibility and between-run reproducibility over time using quality control reagents. The analyser was also found to be sufficiently accurate for detecting pathological values in patients (age > 10 years) [40].

Though not strictly POCT, CityAssays requires a dried blood spot from a finger prick blood sample and is designed for use by the patient directly for home testing, with a reported turnaround time of 3 days for results. Dried blood spots obtained through unsupervised sampling of participants at home have been reported in the literature as a viable methodology for obtaining vitamin D status information [41]. Thus, as with the other devices tested, although there are limitations when compared with the reference standards, there may be benefit in community settings to identify high risk individuals in need of formal testing, diagnosis, and onward management.

The growing burden of NCDs is widely documented in the medical literature [1–3] and there is growing support for community based AHPs, such as dental professionals and pharmacists working collaboratively with medical colleagues to facilitate improved early identification of NCDs. The impact of incorporating POCTs into routine care is yet to be fully established. However, POCT may assist in the early identification of patients at risk of NCDS and facilitate prevention strategies. However, further research would be needed to ascertain this and to evaluate the cost-effectiveness of such methods.

AHPs undertaking risk-targeted early case detection for individuals at high risk of NCDs may be a viable option to detect these conditions early, allowing upstream intervention. In the UK, government policy and NICE guidance [9] already exist supporting AHPs contributing to the early detection of certain NCDs. Furthermore, many dentists and pharmacists already use POCT devices thus, studies such as this, highlighting devices that demonstrate good levels of concordance are important to assist allied healthcare professionals who may be considering undertaking such testing. However, it is important to bear in mind the controversies related to such a model including the potential for increasing the number of referrals to a GP service already working at and beyond capacity. It is important that prior to undertaking any targeted risk-based detection AHPs establish whether patients are already being monitored or have been tested elsewhere to avoid duplication of testing. Likewise, it is important to remember that AHPs should not formally diagnose NCDs, nor would they be the healthcare professional best placed to manage these patients once they are formally diagnosed. AHPs would undertake the test as a means of identifying those patients who would benefit from more formal investigation and management from their primary care practitioner. Thus, it is imperative that clear care pathways are developed in conjunction with the appropriate healthcare professionals to ensure that patients identified as high-risk can be directed to the appropriate service for formal diagnosis and management.

Limitations of this present study include the relatively small sample size of approximately fifty patients per device. However, care was taken to ensure an adequate proportion of participants demonstrating a full range of biochemical values across the distribution curve were recruited. A further limitation of the study was that the results are only applicable to the specific devices tested and cannot be generalised to other devices available in the market. Although the vitamin D testing strips utilise a collection method for capillary blood samples, akin to that for other POCT devices, as the sample needs to be posted to the laboratory, the analytical pathway is not strictly a POCT pathway. However, for the purposes of determining feasibility of use by AHPs in community settings, it fulfils the requirements of being practical and feasible and providing results directly to patients within a reasonable timeframe, hence its inclusion in the present study. A further limitation is the subjective nature of VAS scores. It is recognised that patients assess pain subjectively and that there is likely to be considerable variation of pain thresholds amongst patients tested.

## Conclusion

This study provides evidence to support the use of POCT devices in addition to validated risk assessment questionnaires to identify those at increased risk of, or who unknowingly have NCDs. The study contributes to a broader body of work demonstrating support from stakeholders for allied healthcare professionals undertaking risk assessments for NCDs [5, 6, 17]. The POCT methods employed demonstrated high levels of concordance with standard laboratory methods. Thus there is potential for POCT devices to be used as screening tools leading to further confirmatory tests for formal diagnosis. Further, larger scale studies are however required to determine the effectiveness and cost-effectiveness of POCT devices being used in risk-targeted early detection for NCDs by allied healthcare professionals.

## Data Availability

The datasets used and/or analysed during the current study are available from the corresponding author upon reasonable request.

## Abbreviations

AHPs: Allied Health Professionals
NCDs: Non-communicable disease
POCT: Point of care testing
T2DM: Type-2 diabetes
CKD: Chronic kidney disease
NICE: National Institute for health and Care Excellence
NDH: Non-diabetic hyperglycaemia (Prediabetes)
PIL: Patient information leaflet
VAS: Visual analogue score
SOP: Standard operating procedure
BDH: Birmingham’s Dental Hospital
QEH: Queen Elizabeth Hospital
QC: Quality control
BA: Bland–Altman
